# The Vaginal Microbiome: A Long Urogenital Colonization Throughout Woman Life

**DOI:** 10.3389/fcimb.2021.686167

**Published:** 2021-07-06

**Authors:** Renata S. Auriemma, Roberta Scairati, Guendalina del Vecchio, Alessia Liccardi, Nunzia Verde, Rosa Pirchio, Rosario Pivonello, Danilo Ercolini, Annamaria Colao

**Affiliations:** ^1^ Dipartimento di Medicina Clinica e Chirurgia, Università Federico II di Napoli, Naples, Italy; ^2^ Department of Agricultural Sciences, University Federico II, Naples, Italy; ^3^ United Nations Educational, Scientific and Cultural Organization (UNESCO), Chair for Health Education and Sustainable Development, University Federico II, Naples, Italy

**Keywords:** vaginal microbiome, woman homeostasis, estrogen and progestin variations, bacterial vaginosis, woman health

## Abstract

Vaginal microbial niche is a dynamic ecosystem, composed by more than 200 bacterial species which are influenced by genes, ethnic background and environmental-behavioral factors. Several lines of evidence have well documented that vaginal microbiome constantly changes over the course of woman’s life, so to exert an important impact on woman quality of life, from newborn to post-menopausal ages. This review aims at analyzing the role of vaginal microbiome in the maintenance of woman’s homeostasis and at tracking critical changes that commonly occur across woman’s lifetime. The role of hormone replacement therapy in the modulation of vaginal microbiome composition and in the improvement of vaginal wellness in postmenopausal women with decreasing levels of circulating estrogen is discussed.

## Introduction

The vaginal microbiome is a complex ecological system including commensal, symbiotic and pathogenic organisms that inhabit the vaginal surfaces and its cavity, while maintaining its own homeostasis through mutualistic relationships with the host (Sobel, 1999). This habitat is normally dominated by a limited number of species of lactic acid bacteria (LAB) variously represented in the different reproductive-aged women, which act collectively against various diseases, such as infectious, urogenital, and metabolic diseases (Ravel et al., 2011).

The habitat is strongly interconnected to subject race or ethnicity as well as personal and social behaviors, such as dietary habits, use of probiotics, hygiene and sexual practices, or use of lubricants (Durán et al., 2020).

Recent efforts of culture-independent metagenomics approaches have identified the microbial communities populating the vagina (Relman, 2015). Particularly, species of *Lactobacillus* spp. are dominant in most women, playing a crucial role in both vaginal health and gynecologic wellness (Redondo-Lopez et al., 1990; Witkin et al., 2007; Martin, 2012). LAB are able to generate hydrogen peroxide and lactic acid, to create an acidic microenvironment in response to estrogen levels variations in the bloodstream, to closely adhere onto specialized epithelium, while through competitive exclusion restraining the growth of most pathogens, widely implicated in various gynecological disorders (Redondo-Lopez et al., 1990; Witkin et al., 2007; Martin, 2012). When LAB levels are decreased, the anaerobic bacteria can occur and often determine bacterial vaginosis. In the culture-independent assessment of human vaginal microbiome, *Prevotella* was found as one of the dominant genera (Diop et al., 2019). *Prevotella* are among the most prevalent bacteria in the human microbiome, and their diversity and potential roles have been recently evaluated (De Filippis et al., 2019). In particular, *P. bivia* is linked to epithelial cytokine production, identified in infected biological samples during pregnancy complications. In the vagina, some examples of synergistic relationships occur, such as the case of *P. bivia* and *Gardnerella vaginalis* (Randis and Ratner, 2019).

The vaginal microenvironment is exposed to substantial modifications throughout women life. Several stressors, such as hormones and habits, can dramatically change vaginal microbiome composition, resulting for example in the depletion of LAB, reduction of microbial diversity and occurrence of dysbiosis, often leading to bacterial vaginosis, yeast infections, sexually transmitted diseases, urinary tract infections and poor fertility (Pybus and Onderdonk, 1999; Donders et al., 2000; Marrazzo, 2006). Dietary intake has been shown to play a role in influencing the composition of vaginal microenvironment in women of childbearing age, since a lower concentration of some essential nutrients, such as vitamins A, C and E, b-carotene deficiency and an altered iron status may contribute to the risk of bacterial vaginosis and Candida colonization. At a similar extent, increased plasma glucose levels and dietary fat, as well as obesity, are associated with poorer vaginal health and dysbiosis state (Durán et al., 2020). Nevertheless, a recent prospective cohort study investigating the link between BMI and incident bacterial vaginosis has suggested that obese women had a lower risk of bacterial vaginosis compared to women with normal BMI (Lokken et al., 2010). Similarly, smoking habits have been found associated with bacterial vaginosis, due to a lower *Lactobacilli* count and higher Nugent scores (Durán et al., 2020).

A better understanding of vaginal microbiome composition, functions and changes may be pivotal in gaining new pathophysiological insights in women’s diseases that could be used to implement strategies of prevention.

Here we provide a survey of the vaginal microbiome composition throughout the entire woman life, considering that its changes from birth, throughout puberty, reproductive age and menopause. In addition, we highlight how changes in microbiome composition may critically impact woman health.

## From Newborn to Menarche

**Figure 1:**
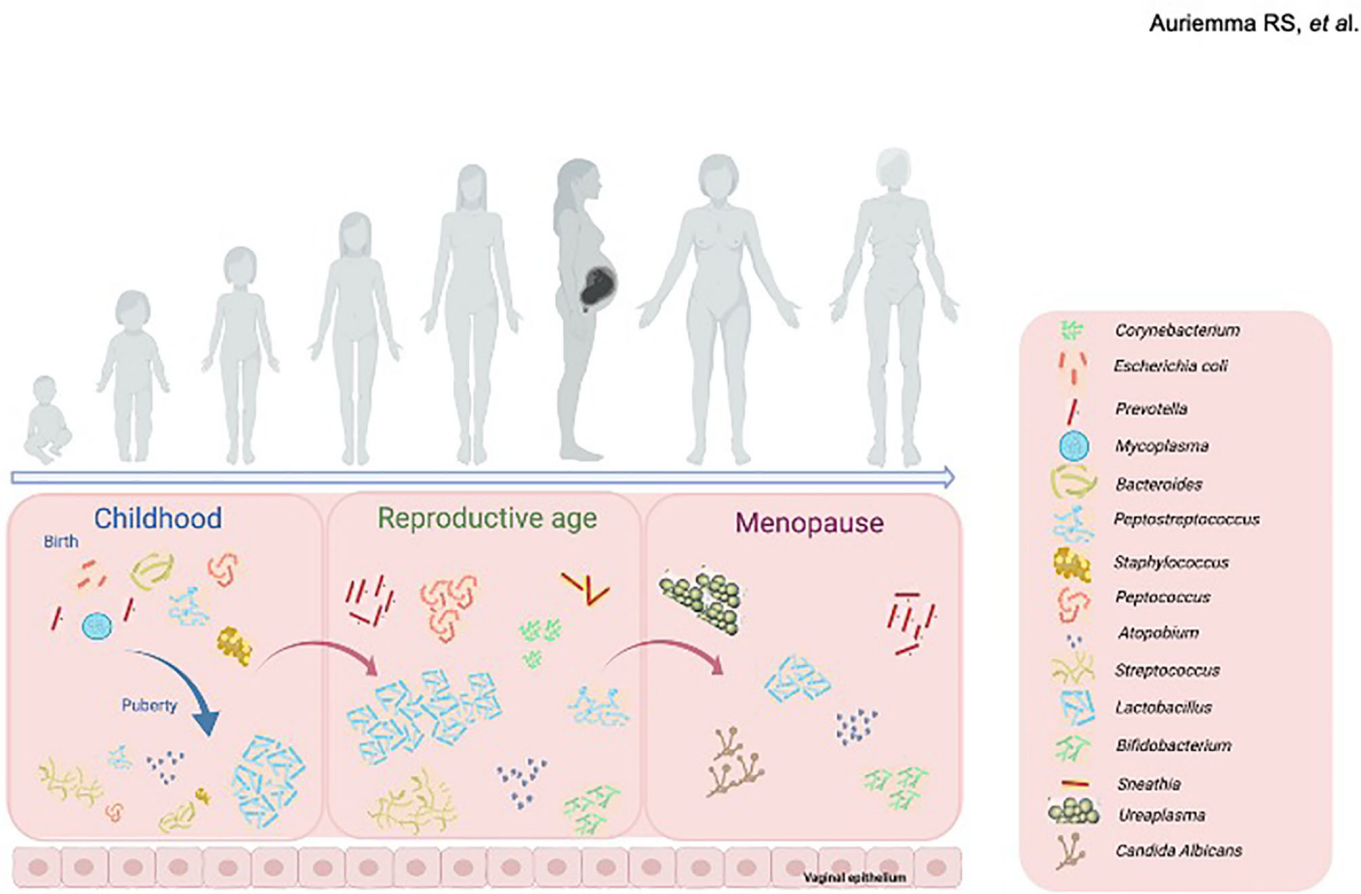
Changes in the composition of vaginal microbiome throughout women life. In childhood (left panel), *Corynebacterium* spp., coagulase-negative *Staphylococcus* spp., *Escherichia coli* and *Mycoplasma* spp., form the vaginal microbiome. At puberty, upon estrogen/progestin exposure the vaginal microbial niche shifts towards other predominant colonies, mainly including *Lactobacillus* spp., *Atopobium*, and *Streptococcus* spp. During reproductive age (middle panel), vaginal microbiome houses a range of bacterial communities, mainly including lactobacilli (*L. crispatus, L. gasseri, L. jensenii and L. iners*) along with anaerobic bacteria. Menopause (right panel) induces further changes in the composition of vaginal microbiome, mainly composed by *Gardnerella vaginalis*, *Ureaplasma urealyticum*, *Candida albicans* and *Prevotella* spp., together with a progressive decrease in species of *Lactobacillus*. Created with BioRender.com.

Several bacteria species constitute the vaginal microbiome in early childhood (**Figure 1**), including anaerobes, *Diphtheroids* (particularly *Corynebacterium* spp.) coagulase-negative *Staphylococci*, *E. coli* and *Mycoplasma* spp (Hammerschlag et al., 1978a; Hammerschlag et al., 1978b). Conversely, upon estrogen/progestin exposure with puberty (**Figure 1**), the vaginal microbial niche shifts towards other predominant colonies, as *Lactobacillus* spp., *Atopobium*, and *Streptococcus* spp (Durán et al., 2020).

Historically, it has been thought that the fetus develops in a sterile environment during pregnancy (Tamburini et al., 2016). However, recent next-generation sequencing studies from placental and fetal samples have challenged the hypothesis of a sterile womb, as microbial DNA fragments have been found in the uterine environment (Blaser et al., 2021). These intriguing results have raised the questions of whether the presence of an indigenous resident and growing microbial community in utero should be postulated, or whether it results from a systemic trafficking of bacterial metabolites during pregnancy and at delivery, and what are potential functional consequences on fetal development (Blaser et al., 2021).

To date, scientific evidence does not truly support an in utero microbial colonization, and the vast majority of human studies confirm the hypothesis of a sterile womb but contemplate the possibility of a transient exposure during gestation and at the time of delivery (Blaser et al., 2021).

Furthermore, recent studies have established an higher stability of vaginal microbial composition, mostly represented by *Lactobacillus* spp. earlier in pregnancy. On the other side, the occurrence of vaginal infections during pregnancy may induce a more diverse and lesser stable vaginal microbiome, to alter uterine environment. Several studies have shown that about 25% of preterm infants were born from mothers with intrauterine infections, which might have eventually contributed to the onset of preterm labor, suggesting a possible direct link between vaginal microbiome composition and early life outcomes (Goldenberg et al., 2008; Aagaard et al., 2014; Romero et al., 2014; Tamburini et al., 2016; Freitas et al., 2018). For instance, the presence of common vaginal residents such as *Burkholderia* and *Lactobacillus iners* has been found associated with preterm delivery (Aagaard et al., 2014), whereas the presence of *A. vaginae* and *Leptotrichia* spp. is commonly linked with dysbiotic state (Romero et al., 2014). However, these results deserve further and more accurate investigations, due to the methodological and technical concerns of the research works taken into account.

While different factors, including delivery mode, environmental exposures, antibiotics, breast feeding, probiotics and prebiotics intake, and genetic aspects, have been associated with gut microbiome changes in early life, very little is known about the role of these determinants on the vaginal microbial composition (Tamburini et al., 2016).

In newborns vaginal microbiome is mainly influenced by the presence of transplacental estrogens for glycogen supply, which is normally metabolized by human α-amylase in maltose and maltotriose used by LAB as metabolic substrate to produce lactic acid (Durán et al., 2020). Therefore, the sudden cessation of transplacental estrogens has been reported to reduce vaginal glycogen content, so that the resulting vaginal pH is neutralized or alkalized (Miller et al., 2016).

In childhood and pre-pubescent stages, vaginal pH remains neutral or alkaline, and is influenced by the constant presence of aerobic, strictly anaerobic and enteric species of bacteria (Donders et al., 2000; Marrazzo, 2006; Romero et al., 2014) whereas LAB are only sporadically found (Hammerschlag et al., 1978a; Hammerschlag et al., 1978b; Thoma et al., 2011). Later on, puberty onset induces a rise in estrogens and progesterone levels with a re-shaping of the vaginal microbiome, thereby favoring LAB colonization throughout the reproductive age of women. A comprehensive study by Eschenbach et al. (2000) has demonstrated that vaginal microbiome changes also with hormonal fluctuations commonly happening during the menstruum. Particularly, in healthy women without bacterial vaginosis, it was found that *Lactobacillus* spp. predominantly increased while *Prevotella* sp. decreased from day 1 to day 24 of the menstrual cycle (Eschenbach et al., 2000).

Whether the maternal microbiome environment might influence health status after birth and during childhood is yet to be fully elucidated. The importance of maternal-newborn sharing of essential microbes to support early life stages opens to the usage of probiotics supplementation in newborns (Braegger et al., 2011); in this regard, *Lactobacillus* and *Bifidobacterium*-enriched supplements have been proven effective in the treatment of atopic dermatitis (Panduru et al., 2015) and infantile colitis (Savino et al., 2010), presumably because of their ability to remodel the gut microbiome; however, no evidence of functional effects on vaginal microbiome have been ever reported. Thus, based on these preliminary observations, it would be highly intriguing to understand whether and how probiotics may influence the vaginal microbiome (beyond the gut system) and also protect against various diseases of the newborn.

## Reproductive-Aged Women

In reproductive-aged women, the vaginal microbiome houses a range of bacterial communities, including at most species of the genus *Lactobacillus* (*L. crispatus*, *L. gasseri*, *L. jensenii* and *L. iners*) (Vasquez et al., 2002; Fredricks et al., 2005), anaerobic bacteria and variably variable amounts other less common bacteria (e.g. *Atopobium*, *Prevotella*, *Parvimonas*, *Sneathia*, *Gardnerella*, *Mobiluncus* and *Peptoniphilus*), which are more frequently observed in bacterial vaginosis (Coolen et al., 2005; Marrazzo, 2011).

Across reproductive age, the vaginal microbiome is exposed to estrogens and progestin, and some crucial changes may happen, such as a lowering of local pH (<4.5) due to glycogen metabolization, which in turn may restrict the growth of many pathogens and even induce structural modifications of the vaginal epithelium (Farage and Maibach, 2006). In fact, as soon as reproductive age begins the rise in estrogens promotes the vaginal mucosal epithelium hyperplasia and increases cellular glycogen content (Amabebe and Anumba, 2018). Glycogen is catabolized by human α-amylase to maltose, maltotriose and α-dextrines, then further metabolized to lactic acid by *Lactobacillus* species (Amabebe and Anumba, 2018). Consequently vaginal pH reaches 3.5-4.5, which is suitable for the adhesion, colonization and survival of *Lactobacillus* and other bacterial species (Amabebe and Anumba, 2018). Therefore, estrogens play an important role on both vaginal microbiome composition and vaginal epithelium: the progressive increase in estrogen levels from puberty to reproductive age triggers the transition from low glycogen levels, high microbial diversity, high vaginal pH and thin vaginal epithelium during puberty, to high glycogen deposits in the epithelial cells and free glycogen available for *Lactobacillus* spp., which then predominates the healthy vaginal microbiome, and thickening of vaginal epithelium (Amabebe and Anumba, 2018).

An imbalance in the composition of vaginal microbiome across this age, as it commonly occurs in dysbiosis, may result in bacterial vaginosis, an infectious process that is clinically defined when three out of four Amsel’s criteria are fulfilled (Amsel et al., 1983): i) excessive white vaginal discharge; ii) fishy malodor; iii) vaginal pH secretion > 4.5; iv) “clue” cells in wet preparations. A laboratory scoring system, namely Nugent score (Nugent et al., 1991), based on the quantitative assessment of *Lactobacillus* and other microroganisms (*Gardenerella vaginalis, Prevotella* spp.*, Mobiluncus*) on Gram-stained vaginal smear, allows a more sensitive detection of bacterial vaginosis as compared to clinical criteria only (Amsel et al., 1983). Indeed, high LAB levels are associated with a healthy state, whereas their depletion along with the prevalence of other morphotypes are more likely observed in bacterial vaginosis (Nugent et al., 1991). For a long time, high levels of *Lactobacillus* at vaginal level in reproductive-aged women has been thought to be healthy, whereas a low or absent concentration has been recognized as non-optimal state (Priestley et al., 1997). The assumption that LAB cooperatively work for the maintenance of a healthy vaginal environment mainly relies on *in vitro* bacteria cultures studies which, albeit limited by techniques-dependent variables, have advanced the scientific knowledge on vaginal microbiome composition over the years (Hay et al., 1997; Keane et al., 1997; Schwebke et al., 1999).

However, the advent of culture-independent methods, such as 16S rRNA gene sequencing (Stackebrandt and Goebel, 1994), has led to comprehensively capture resident bacteria taxa in the vagina (Bakken, 1985; Ravel et al., 2011; Pasolli et al., 2020). Indeed, by performing means of 16S rRNA gene sequencing, a pioneering study by Ravel et al. (2011), performed on a cohort of 396 asymptomatic and sexually active women of four different ethnicities, has found a myriad of resident bacteria species, and has proposed to classify five main vaginal microbial subtypes, namely community state types (CSTs). Based on the 282 taxa identified, patients were grouped into 5 distinct community clusters. CSTs I, II, III and V, commonly associated with a healthy vagina, found in 73% of women, were dominated by different species of *Lactobacilli* (*L. iners, L. crispatus, L. gasseri*, or *L. jenseni*), and, more likely, were mainly represented among Asian and White communities (Ravel et al., 2011). A recent large-scale analysis of LAB genomes from food and human microbiomes indicated that *L. crispatus, L. gasseri* and *L. jenseni* are indeed the most prevalent in vagina samples (Turnbaugh et al., 2007). CST IV, further subclassified as IVA and IVB, characterized by a lower amount of LAB but higher proportions of strictly anaerobic organisms (*Gardnerella, Atopobium, Mobiluncus, Prevotella* and *Leptotrichia*), clustered more within Hispanics and Blacks, and were linked to a dysbiosis state, high Nugent score and an asymptomatic un-healthy state (Ravel et al., 2011). These findings, later confirmed in other investigations (Gajer et al., 2012), have demonstrated that LAB may have a crucial role in the maintenance of a healthy vaginal microbiome. Moreover, these results have also shown that interindividual variability does exist and most likely relies on complex interactions between microbial species and vaginal environment. In both studies (Turnbaugh et al., 2007; Ravel et al., 2011) each community cluster has been reported to be relatively stable over time in some cases, whereas, in other cases, a shift among CSTs has been observed and associated with a high microbial turnover. However, women shifting their CST otherwise had a good acidic vaginal environment, performed well on Nugent (low score) and kept a healthy vaginal state (Ravel et al., 2011; Gajer et al., 2012). Among *Lactobacillus* species, *L. iners* is reported to have a controversial role in maintaining vaginal ecosystem. Its genome encodes proteins involved in the optimal adaptation to the vaginal niche, such as iron-sulfur proteins and unique σ-factors. However, *L. iners* has also been isolated from vaginal microbiota of bacterial vaginosis, suggesting that this species can easily adapt to the fluctuating vaginal niche or lead to a symbiotic or parasitic lifestyle. *L. iners-*dominated bacterial vaginosis may be also associated with higher risk of preterm labor, endometritis or other reproductive tract infections (Petrova et al., 2017).

Adding complexity on vaginal microbiome heterogeneity, several studies have also included woman race as a confounding variable and have thus reported contrasting results on CST-race specificity. In fact, each distinct CST could be easily found across some human races (Japanese, White and Black North American women), and, as a result, vaginal microbiome composition might be influenced by genetic/immunological cues more likely than social/behavior habits (Zhou et al., 2007). Moreover, a given CST could associate with a given race (Zhou et al., 2007), as for instance women of European Ancestry more easily carry a *Lactobacilli*-dominated microbiome, whereas Hispanic and African American women do not (Fettweis et al., 2014).

Altogether these findings suggest that the rise in sexual hormones, both estrogens and progestin, after menarche and during reproductive age induces crucial changes in the composition of vaginal microbiome, which contributes to the maintenance of a healthy vaginal environment. In this respect, LAB play a crucial role for vaginal homeostasis, and the imbalance toward other microbial species may prompt the development of un-healthy states. However, vaginal microbiome composition is very heterogeneous among individuals, as it is influenced by genetic and immunological factors as well as ethnicity, besides social habits and hormone levels.

Vaginal microbiome reportedly plays a crucial role also in human papillomavirus (HPV) infections, known to be associated with cervical carcinoma progression. Vaginal microbiota diversity may be involved in regulating HPV persistence and cervical intraepithelial neoplasia (CIN) disease severity (Mitra et al., 2015). Particularly, low grade squamous intra-epithelial lesions (LSIL) samples have shown a significant over-representation of *Lactobacillus jensenii* and *Lactobacillus coleohominis*; high grade squamous intra-epithelial lesions (HSIL) samples were found to have significantly higher levels of *Peptostreptococcus anaerobius*, *Anaerococcus tetradius* and a significant over-representation of *Fusobacteria* and *Sneathia sanguinegens*. Increasing disease severity was also associated with decreasing relative abundance of *Lactobacillus* spp (Mitra et al., 2015). Vaginal samples exhibiting a low relative abundance of *Lactobacillus* spp. (as in CST IV) or dominated by *L. iners* (CST III) had the major proportion of HPV-positive samples, suggesting an increasing risk of HPV acquisition and/or persistence in these two states (Brotman et al., 2014). Conversely, women with vaginal microbiota dominated by *L. gasseri* (CST II) had a faster HPV remission rate (Brotman et al., 2014). A Korean study has identified a diversity of vaginal microbiota among monozygotic twin women depending on the presence or absence of HPV. A lower presence of *Lactobacilli* spp. has been found in HPV-positive women, denoting the presence of *Sneathia* s*pp* as risk factor for HPV infection (Lee et al., 2013). Consistently, CST IV seems to be associated with HPV infection, being characterized by a greater diversity of species than other CSTs, with a lower presence of *Lactobacilli* spp. in favor of anaerobes such as *Gardnerella*, *Megasphera*, *Sneathia* and *Prevotella* (Romero et al., 2014). The increased growth of these anerobic species determines bacterial vaginosis, a condition that has been associated with the infection and persistence of HPV and the consequent development of CIN (Guo et al., 2012).

Therefore, at vaginal level, unlike other districts of the body, microbial variability is associated with a disease state and not with healthy state. The ability to maintain an acidic environment and to produce bacteriocin explains the importance of the predominance of *Lactobacillus* spp. at the vaginal level. Vaginal mucosa is thus intact and protected from the invasion of other microorganisms such as HPV. In fact, in a population of 9165 premenopausal women a 10-20% significant increase in the incidence of HPV infection has been found associated to a pH greater than 5 at the vaginal level (King et al., 2011). Furthermore, the production of bactericidal molecules makes *Lactobacillus* spp. a worthy protector of the vaginal environment. In fact, *L. gasseri*, as well as *L. crispatus* and *L. reuteri* are capable of producing Gasserin which acts on both Gram positive and Gram negative bacteria (Clarke et al., 2012).

However, the most common bacterial sexually transmitted infection worldwide is represented by *Chlamydia trachomatis* (CT), a Gram-negative obligate intracellular bacterial pathogen able to cause simple urogenital tract infections, including asymptomatic urogenital infections such as urethritis and cervicitis, potentially evolving in more serious problems if not promptly treated, such as pelvic inflammatory disease, ectopic pregnancy and infertility (Mitra et al., 2016; WHO, 2018).

Vaginal ecosystem changes during common infections of the female genital tract has been recently investigated by analyzing vaginal microbiome and metabolome in 79 reproductive aged women, by means of next generation sequencing and proton based-nuclear magnetic resonance spectroscopy (Ceccarani et al., 2019). Patients were classified as vulvovaginal candidiasis (VVC, no.18), *Chlamydia trachomatis* infection (CT, no.20), bacterial vaginosis (BV, no.20) and healthy controls (HC, no.21) (Ceccarani et al., 2019). *Lactobacillus* genus was profoundly reduced in all genital infections; species-level analysis revealed that healthy vaginal microbiome was dominated by *L. crispatus*, whereas the shift from HC to CT, VVC, and BV induced the progressive replacement from *L. crispatus* to *L. iners* (Ceccarani et al., 2019). VVC, CT and BV have been reported to be characterized by anaerobe genera, including. *Gardnerella*, *Prevotella*, *Megasphaera*, *Roseburia* and *Atopobium* (Ceccarani et al., 2019). These bacterial communities changes during the genital infections resulted in the significant decrease of lactate and the pathological alteration in the vaginal metabolites composition (Ceccarani et al., 2019). These findings reinforce the evidence that the vaginal microbiome is an important first line defense for several types of infections and its modifications can favor the invasion by microorganisms. In healthy women microbiome abounds in *L. crispatus* that has been shown to be a protective factor for CT adhesion, unlike *L. iners* (Ceccarani et al., 2019). The former has a greater capacity to produce lactic acid both L and D dimers and H2O2, allowing a more acidic environment that can counteract the action of CT by destruction of the surface molecule(s), destruction of the membrane, and disruption of the internal metabolism (Boskey et al., 1999; Nardini et al., 2016). Indeed a case control study has shown that *L. iners*-dominated community is a risk factor for CT infection (Houdt et al., 2018). Another microorganism connected with CT infection is *Prevotella*, as it is a source of tryptophan, an amino acid essential for CT growth (Ziklo et al., 2016). Conversely, *Lactobacillus* concentration was found not significantly changed during VVC, resulting from the excessive growth of *Candida* species normally colonizing the vaginal environment (Underhill, 2013). In fact, during VVC vaginal pH is reported normal (Workowski and Berman, 2010), and a *Lactobacilli* dominant microbiota has been found in patients with VVC (Wu et al., 2017). These data are confirmed in other studies, reporting no difference in vaginal microbiome composition between healthy woman and those with severe VVC (Zhou et al., 2009; Vylkova et al., 2011), and no typical pattern of microbiota during VVC has been identified (Liu et al., 2013).

Whether the use of probiotics might be effective for treatment of dysbiosis in reproductive aged women is still under investigation. Some studies have proposed an emerging role for prebiotics and probiotics in the remodeling of the vaginal microenvironment in women of childbearing age (Huang et al., 2014; Xie et al., 2017). In this respect, a recent Cochrane metanalysis (**Table 1**, Xie et al., 2017) analyzed the role of probiotics supplements in a large patient cohort of 1656 non-pregnant women affected by vulvo-vaginal candidiasis. This comprehensive study revealed that the combination of probiotics with antifungals may represent an effective strategy against this infectious disease, compared to the only administration of conventional therapeutics with antifungal drugs (Xie et al., 2017); in addition, combined treatment with antifungals and probiotics supplements, taken by oral and vaginal routes, could improve short-term clinical and mycological cure rates without influencing long-term outcomes and decrease the rate of relapse at 1 month follow-up. Similarly, a large metanalysis including 12 randomized controlled trials for a total of 1,304 participants has demonstrated some satisfactory results of probiotics against bacterial vaginosis with a significant improvement of cure rate after 21 and 30 days of treatment (**Table 2**, Huang et al., 2014). However, these results deserve further and more accurate investigations, due to the overall low-quality evidence of the research works taken into account.

**Table 1:** Effects of probiotics supplementation in non-pregnant women affected by vulvo-vaginal candidiasis.

List of research studies included in the Cochrane metanalysis on the usage of probiotics added to antifungals for the treatment of vulvo-vaginal candidiasis (patient cohort = 1656 women)			
Study	Therapeutic regimen	Route of probiotics administration	Endpoints
*Zhang 2005* (Turnbaugh et al., 2007)	L. delbrueckii subsp. Lactis DM8909 + Miconazole	Vaginal	Long-term clinical and mycological cure rates (assessed at 1 and 3 months after completion of the treatment); Rate of adverse events.
*Han 2006* (Gajer et al., 2012)	L. delbrueckii subsp. Lactis DM8909 + Clotrimazole	Vaginal	Short-term clinical and mycological cure rates (assessed at 7-10 days after completion of treatment); First relapse after treatment (1 month after completion of treatment); Rate of adverse events.
*Mai 2007* (Petrova et al., 2017)	L. delbrueckii subsp. Lactis DM8909 + Clotrimazole	Vaginal	Short-term mycological cure rate (7 days after completion of treatment); Rate of adverse events.
*Hua 2008* (Zhou et al., 2007)	L. delbrueckii subsp. Lactis DM8909 + Miconazole	Vaginal	Short-term clinical and mycological cure rate (5-7 days after completion of treatment); First relapse after treatment (33-37 days after completion of treatment)
*Yang 2009* (Fettweis et al., 2014)	L. delbrueckii subsp. Lactis DM8909 + Clotrimazole	Vaginal	Short-term clinical and mycological cure rate (7-10 days after completion of treatment); First relapse after treatment (1 month after completion of treatment); Rate of adverse events.
*Ma 2007* (Mitra et al., 2015)	Streptococcus faecalis + Miconazole	Vaginal	Short-term clinical and mycological cure rate (7 days after completion of treatment); Rate of adverse events.
*Lin 2006* (Brotman et al., 2014)	Streptococcus faecalis + Clotrimazole	Vaginal	Short-term mycological cure rate (7 days after completion of treatment); Rate of adverse events.
*Martinez 2009* (Lee et al., 2013)	L. rhamnosus GR-1 and L. reuteri RC-14 + Fluconazole	Oral	Long-term mycological cure rate (28 days after beginning of treatment).
*Nouraei 2012* (Romero et al., 2014)	L. casei, L. rhamnosus, S. thermophilus, Bifidobacterium breve, L. acidophilus, Bifidobacterium longum and L. bulgaricus + Fluconazole	Oral	Short-term clinical cure rate; Short-term mycological cure rate.
*Kovachev 2015* (Guo et al., 2012)	L. acidophilus, L. rhamnosus, S. thermophilus and L. delbrueckii subsp. Bulgaricus + Fluconazole plus Fenticonazole	Fluconazole: oral Fenticonazole: vaginal Probiotics: vaginal	Long-term mycological cure rate (28 days after beginning of treatment)

Adapted from reference (Pasolli et al., 2020).

**Table 2:** Effects of probiotics supplementation in non-pregnant women affected by bacterial vaginosis.

List of research studies included in the Huang metanalysis on the usage of probiotics alone or as adjuvants against bacterial vaginosis (patient cohort = 1304 women)			
Study	Therapeutic regimen	Route of probiotics administration	Risk Ratio [95% confidence interval (CI)] of the proposed therapeutic regimen compared to placebo
*Hallen 1992* (King et al., 2011)	L. acidophilus	Vaginal	34.14 [2.15, 543.06]
*Parent 1996* (Clarke et al., 2012)	Hydrogen peroxide-producing strain of L. acidophilus + Estriol	Vaginal	3.50 [1.47, 8.34]
*Eriksson 2005* (Mitra et al., 2016)	L. gasseri, L. casei rhamnosus, L. fermentum + Clindamycin	Vaginal	0.90 [0.71, 1.14]
*Anukam 2006* (WHO, 2018)	L. rhamnosus gR-1 and L. reuteri RC-14 + Metronidazole	Oral	2.19 [1.59, 3.03]
*Petricevic 2008* (Ceccarani et al., 2019)	L. casei rhamnosus (Lcr35), lactose, and magnesium stearate + antibiotic treatment	Vaginal	2.32 [1.75, 3.09]
*Marcone 2008* (Nardini et al., 2016)	Freeze-dried L. rhamnosus + Metronidazole	Vaginal	1.09 [0.91, 1.31]
*Larsson 2008* (Boskey et al., 1999)	Freeze-dried lactobacilli + Clindamycin cream	Vaginal	1.43 [1.00, 2.06]
*Mastromarino 2009* (Houdt et al., 2018)	L. brevis CD2, L. salivarius FV2, and L. plantarum FV9	Vaginal	8.00 [1.13, 56,41]
*Martinez 2009* (Ziklo et al., 2016)	L. rhamnosus GR-1 and L. reuteri RC + Tinidazole	Vaginal	1.75 [1.21, 2.53]
*Ya 2010* (Underhill, 2013)	Lactobacillus rhamnosus, L acidophilus, and Streptococcus thermophilus	Vaginal	1.53 [1.19, 1.98]
*Marcone 2010* (Workowski and Berman, 2010)	L. rhamnosus + Metronidazole	Vaginal	1.24 [0.94, 1.62]
*Bradshaw 2012* (Wu et al., 2017)	L. acidophilus KS400, Estriol + Metronidazole	Vaginal	0.95 [0.82, 1.11]
			**Total risk ratio (RR)** = 1.53; 95% confidence interval (CI) 1.19–1.97

Adapted from reference (Bakken, 1985).

Similarly to the successful use of fecal microbiota transplantation in intestinal infections, recently the potential role of vaginal microbiome transplantation (VMT) from healthy donors has been investigated as a therapeutic alternative for patients with symptomatic and recurrent bacterial vaginosis (Lev-Sagie et al., 2019). Four out of five treated patients had a full long-term remission up to last follow-up at 5-21 months after VMT, associated with the reconstitution of a *Lactobacillus*-dominated vaginal microbiome (Lev-Sagie et al., 2019).

## Menopause

A wide range of vaginal microbiome composition has been shown in menopausal age, and relative abundance of microbial species in the vagina largely varies among women (Srinivasan et al., 2010; Brotman et al., 2014). Moreover, vaginal microbiome has been reported to play a crucial role in postmenopausal women mainly because it may exert profound effects on the development of vulvovaginal atrophy, vaginal dryness and impaired sexual health (Muhleisen and Herbst-Kralovetz, 2016).

Generally, the decrease in LAB levels in postmenopausal women is considered a physiologic change (Muhleisen and Herbst-Kralovetz, 2016). In 1997 Hillier et al. investigated vaginal microflora in 73 postmenopausal women, who had not received hormonal replacement therapy (HRT) (Hillier and Lau, 1997). *Lactobacilli* were detected in 49% of women, whereas *Gardnerella vaginalis* was found in 27%, *Ureaplasma urealyticum* in 13%, *Candida albicans* in 1%, and *Prevotella bivia* in 33% of women (Fettweis et al., 2014). This study concluded that species of *Lactobacillus* and other vaginosis-associated bacteria were less common in postmenopausal woman than those found in women at fertile age (Hillier and Lau, 1997). Similarly, the prevalence of bacterial vaginosis has been reported overall lower in postmenopausal women (6.0%) than in fertile (9,8%) and perimenopausal (11%) woman (Cauci et al., 2002). Particularly, 6.3% of postmenopausal women not receiving HRT and 5.4% of those treated with HRT have been found positive for bacterial vaginosis (Cauci et al., 2002). Therefore, the reversion of LAB microbial diversity to premenopausal levels following HRT has been shown not to increase the prevalence of bacterial vaginosis in postmenopausal women (Cauci et al., 2002).

The composition of the vaginal microbiome has been investigated in a cohort of 87 women, including 30 premenopausal, 29 perimenopausal, and 28 postmenopausal women (Brotman et al., 2018). Bacterial communities were grouped into six state types (CST), including *Lactobacillus crispatus* in CST I, *Lactobacillus gasseri* in CST II, *Lactobacillus iners* in CST III, *Lactobacillus jensenii* in CST V*, Streptococcus* and *Prevotella* in CST IV-A, and *Atopobium* in CST IV-B (Liu et al., 2013). A significant association was found between CSTs and menopause stage, as well as between CSTs and vulvovaginal atrophy (Brotman et al., 2018).

A recent study by Cohen et al. (2020) has suggested that the administration of *Lactobacillus crispatus* strain CTV-05 could colonized women and reduce bacterial vaginosis, when given as s vaginal dose.

In 2015 an American study has analyzed the difference between vaginal glycogen content and *Lactobacillus* species levels in cervicovaginal lavage samples of 11 premenopausal and 12 postmenopausal women (Mirmonsef et al., 2015). Postmenopausal women have been found to display significantly lower levels of free glycogen than premenopausal women, which in turn had higher *Lactobacillus* levels and lower vaginal pH (median pH= 4) than postmenopausal women (median pH= 4.6) (Lee et al., 2013). In all samples, a positive correlation between *L. iners* and glycogen levels has been shown in samples from both premenopausal and postmenopausal women. Similarly, *L. jensenii* levels were significantly associated with free glycogen in all samples. *L. iners* levels were found higher in samples from premenopausal women, whereas *L. crispatus* and *L. jensenii* levels were not significantly different between two groups (Mirmonsef et al., 2015).

However, the most common type of vaginitis in postmenopausal women is aerobic vaginitis (AV), characterized by *Streptococcus spp* as the dominant bacteria. AV induces severe depression of lactate production with clinical features notably different from those of bacterial vaginosis. In fact, AV elicits an important host response with cytokines production and vaginal leucocytes presence (Donders et al., 2002). Usually the color of the discharge in bacterial vaginosis is whitish or gray and of a watery consistency, whereas in AV it is yellow to green and rather thick mucus. Worth to note, severe AV may induce dyspareunia, which is generally not present in women with bacterial vaginosis (Donders et al., 2017).

These findings support that vaginal microbiome composition physiologically changes in postmenopausal women. The fall in estrogen levels results in the decrease of LAB levels, which in turn influences the occurrence of genitourinary menopausal symptoms, mainly including vulvovaginal atrophy, vaginal dryness and impaired sexual health.

The impact of the vaginal microbiome composition on genitourinary menopausal symptoms, serum estrogen, and vaginal glycogen has been investigated in 88 postmenopausal women (Mitchell et al., 2017). Among them, 66% had any *Lactobacillus* species detected and 38% had a *Lactobacillus*-dominant vaginal microbiome (Mitchell et al., 2017). Specifically, 24% of women had both *L. crispatus* and *L. iners*, 9% had *L. crispatus* only, 32% had *L. iners* only, 34% had neither species detected (Mitchell et al., 2017). Genitourinary menopausal symptoms were found not associated with the presence of specific *Lactobacillus* species (Mitchell et al., 2017). Noteworthy, women with *Lactobacillus*-dominant communities were demonstrated to have higher unconjugated serum estrone, but no difference in vaginal glycogen levels as compared to those with non-*Lactobacillus*-dominant communities (Mitchell et al., 2017). However, higher serum estradiol and estrone were not associated with either higher vaginal glycogen (Mitchell et al., 2017).

Whether the use of probiotics might be effective for treatment of postmenopausal symptoms is still under investigation. *Lactobacilli* have been shown to improve vaginal microbiome of menopausal women (Petricevic et al., 2008). Indeed, 72 postmenopausal women with Nugent scores between 4 and 6 were randomized into two groups: 35 women were assigned to the intervention group and received probiotic capsules containing 2.5 10^9^ CFU each of lyophilized *L. rhamnosus* GR-1 and *L. reuteri* RC-14, and 37 women were randomized in the control group and received an oral placebo once daily for 14 days. The median difference in Nugent scores between baseline and the end of the study was 3 in the intervention group and 0 in the control group (Petricevic et al., 2008), thus leading to the substantial improvement in the vaginal flora of postmenopausal women and providing evidence for the use of oral probiotics as a potential alternative approach to restore the normal vaginal flora (Petricevic et al., 2008).

The effect of ultra-low dose vaginal tablets estriol (0.03 mg) and *Lactobacillus acidophilus* compared to placebo have been investigated in 87 postmenopausal women. The instillation of lactobacilli and ultra-low dose estriol was found to significantly improve the vulvovaginal symptoms in such women (Jaisamrarn et al., 2013).

A clinical trial on 60 postmenopausal women (aged 40 to 60 years), randomly assigned to receive oral isoflavone (150 mg dry extract of glycine max) alone, or isoflavone plus probiotic (*L. acidophilus, L. casei, Lactococcus lactis, Bifidobacterium bifidum, *and* Bifidobacterium lactis*), or hormonal therapy (1 mg estradiol and 0.5 mg norethisterone acetate) has demonstrated that after 16 weeks the hormonal therapy group showed an increased number of lactobacilli in the vagina, similar to that seen in premenopausal state, and a decrease vaginal PH; conversely no change in pH value was found in the isoflavone group and isoflavone plus probiotic group (Ribeiro et al., 2018). A recent study by Cohen et al. (2020) conducted on 228 premenopausal suggested that daily administration of vaginal dose of Lactobacillus crispatus,after treatment with vaginal metronidazole, can reduce recurrence of bacterial vaginosis after 12 weeks.

## Author Contributions

RSA conceived the study and supervised the manuscript drafting. RSA, RS, GDV, and AL performed the literature search, contributed to the interpretation of the data, and prepared the figures. RSA, RS, and GDV drafted the work. DE and AC provided significant expert contribution in the scientific content revision process. AC critically reviewed the manuscript and revised it for important intellectual content. All authors contributed to the article and approved the submitted version.

## Conflict of Interest

The authors declare that the research was conducted in the absence of any commercial or financial relationships that could be construed as a potential conflict of interest.

## Abbreviations

LAB, lactic acid bacteria; HRT, hormone replacement therapy; CST, community state types; CFU, colony forming unit.

## References

[B1] Aagaard K. Ma J. Antony K. M. Ganu R. Petrosino J. Versalovic J. (2014). The Placenta Harbors a Unique Microbiome. Sci. Transl. Med. 6 (237), 237ra65. 10.1126/scitranslmed.3008599 PMC4929217 24848255

[B2] Amabebe E. Anumba D. O. C. (2018). The Vaginal Microenvironment: The Physiologic Role of Lactobacilli. Front. Med. (Lausanne) 13 (5), 181. 10.3389/fmed.2018.00181 PMC6008313 29951482

[B3] Amsel R. Totten P. A. Spiegel C. A. Chen K. C. Eschenbach D. Holmes K. K. (1983). Nonspecific Vaginitis. Diagnostic Criteria and Microbial and Epidemiologic Associations. Am. J. Med. 74 (1), 14–22. 10.1016/0002-9343(83)91112-9 6600371

[B4] Bakken L. R. (1985). Separation and Purification of Bacteria From Soil. Appl. Environ. Microbiol. 49 (6), 1482–1487. 10.1128/aem.49.6.1482-1487.1985 16346816 PMC241751

[B5] Blaser M. J. Devkota S. McCoy K. D. Relman D. A. Yassour M. Young V. B. (2021). Lessons Learned From the Prenatal Microbiome Controversy. Microbiome 9 (1), 8. 10.1186/s40168-020-00946-2 33436098 PMC7805060

[B6] Boskey E. R. Telsch K. T. Whaley K. J. (1999). Acid Production by Vaginal Flora In Vitro in Consistent With the Rate and Extent of Vaginal Acidification. Infect. Immunol. 67, 5170–5175. 10.1128/IAI.67.10.5170-5175.1999 10496892 PMC96867

[B7] Braegger C. Chmielewska A. Decsi T. Kolacek S. Mihatsch W. Moreno L.. (2011). Supplementation of Infant Formula With Probiotics and/or Prebiotics: A Systematic Review and Comment by the ESPGHAN Committee on Nutrition. J. Pediatr. Gastroenterol. Nutr. 52 (2), 238–250. 10.1097/MPG.0b013e3181fb9e80 21150647

[B8] Brotman R. M. Shardell M. D. Gajer P. Fadrosh D. Chang K. Silver M. I.. (2018). Association Between the Vaginal Microbiota, Menopause Status, and Signs of Vulvovaginal Atrophy. Menopause 25 (11), 1321–1330. 10.1097/GME.0000000000001236 30358729

[B9] Brotman R. M. Shardell M. D. Gajer P. Tracy J. K. Zenilman J. M. Ravel J.. (2014). Interplay Between the Temporal Dynamics of the Vaginal Microbiota and Human Papillomavirus Detection. J. Infect. Dis. 210 (11), 1723–1733. 10.1093/infdis/jiu330 24943724 PMC4296189

[B10] Cauci S. Driussi S. De Santo D. Penacchioni P. Iannicelli T. Lanzafame P.. (2002). Prevalence of Bacterial Vaginosis and Vaginal Flora Changes in Peri- and Postmenopausal Women. J. Clin. Microbiol. 40 (6), 2147–2152. 10.1128/JCM.40.6.2147-2152.2002 12037079 PMC130764

[B11] Ceccarani C. Foschi C. Parolin C. D’Antuono A. Gaspari V. Consolandi C.. (2019). Diversity of Vaginal Microbiome and Metabolome During Genital Infections. Sci. Rep. 9 (1), 14095. 10.1038/s41598-019-50410-x 31575935 PMC6773718

[B12] Clarke M. A. Rodriguez A. C. Gage J. C. Herrero R. Hildesheim A. Wacholder S.. (2012). A Large, Population-Based Study of Age-Related Associations Between Vaginal pH and Human Papillomavirus Infection. BMC Infect. Dis. 12, 33. 10.1186/1471-2334-12-33 22316377 PMC3292496

[B13] Cohen C. R. Parks T. Hemmerling A. (2020). Randomized Trial of Lactin-V to Prevent Recurrence of Bacterial Vaginosis. N. Engl. J. Med. 383 (8), 791–792. 10.1056/NEJMc2021832 32813960

[B14] Coolen M. J. Post E. Davis C. C. Forney L. J. (2005). Characterization of Microbial Communities Found in the Human Vagina by Analysis of Terminal Restriction Fragment Length Polymorphisms of 16S rRNA Genes. Appl. Environ. Microbiol. 71, 8729–8737. 10.1128/AEM.71.12.8729-8737.2005 16332868 PMC1317315

[B15] De Filippis F. Pasolli E. Tett A. Tarallo S. Naccarati A. De Angelis M.. (2019). Distinct Genetic and Functional Traits of Human Intestinal Prevotella Copri Strains Are Associated With Different Habitual Diets. Cell Host Microbe 25 (3), 444–453.e3. 10.1016/j.chom.2019.01.004 30799264

[B16] Diop K. Dufour J. C. Levasseur A. Fenollar F. (2019). Exhaustive Repertoire of Human Vaginal Microbiota. Hum. Microbiome J. 11, 100051. 10.1016/j.humic.2018.11.002

[B17] Donders G. G. G. Bellen G. Grinceviciene S. Ruban K. Vieira-Baptista P. (2017). Aerobic Vaginitis: No Longer a Stranger. Res. Microbiol. 168 (9-10), 845–858. 10.1016/j.resmic.2017.04.004 28502874

[B18] Donders G. G. Bosmans E. Dekeersmaecker A. Vereecken A. Van Bulck B. Spitz B. (2000). Pathogenesis of Abnormal Vaginal Bacterial Flora. Am. J. Obstet Gynecol. 182 (4), 872–878. 10.1016/S0002-9378(00)70338-3 10764465

[B19] Donders G. G. Vereecken A. Bosmans E. Dekeersmaecker A. Salembier G. Spitz B. (2002). Definition of a Type of Abnormal Vaginal Flora That Is Distinct From Bacterial Vaginosis: Aerobic Vaginitis. BJOG 109 (1), 34–43. 10.1111/j.1471-0528.2002.00432.x 11845812

[B20] Durán A. B. Fuentes-López A. de Salazar A. Plaza-Díaz J. García F. (2020). Reviewing the Composition of Vaginal Microbiota: Inclusion of Nutrition and Probiotic Factors in the Maintenance of Eubiosis. Nutrients 12 (2), 419. 10.3390/nu12020419 PMC7071153 32041107

[B21] Eschenbach D. A. Thwin S. S. Patton D. L. Hooton T. M. Stapleton A. E. Agnew K.. (2000). Influence of the Normal Menstrual Cycle on Vaginal Tissue, Discharge, and Microflora. Clin. Infect. Dis. 30 (6), 901–907. 10.1086/313818 10852812

[B22] Farage M. Maibach H. (2006). Lifetime Changes in the Vulva and Vagina. Arch. Gynecol. Obstet. 273 (4), 195–202. 10.1007/s00404-005-0079-x 16208476

[B23] Fettweis J. M. Brooks J. P. Serrano M. G. Sheth N. U. Girerd P. H. Edwards D. J.. (2014). Differences in Vaginal Microbiome in African American Women Versus Women of European Ancestry. Microbiology 160 (10), 2272–2282. 10.1099/mic.0.081034-0 25073854 PMC4178329

[B24] Fredricks D. N. Fiedler T. L. Marrazzo J. M. (2005). Molecular Identification of Bacteria Associated With Bacterial Vaginosis. N. Engl. J. Med. 353, 1899–1911. 10.1056/NEJMoa043802 16267321

[B25] Freitas A. C. Bocking A. Hill J. E. Money D. M. VOGUE Research Group (2018). Increased Richness and Diversity of the Vaginal Microbiota and Spontaneous Preterm Birth. Microbiome 6 (1), 117. 10.1186/s40168-018-0502-8 29954448 PMC6022438

[B26] Gajer P. Brotman R. M. Bai G. Sakamoto J. Schütte U. M. Zhong X.. (2012). Temporal Dynamics of the Human Vaginal Microbiota. Sci. Transl. Med. 4 (132), 132ra52. 10.1126/scitranslmed.3003605 PMC3722878 22553250

[B27] Goldenberg R. L. Culhane J. F. Iams J. D. Romero R. (2008). Epidemiology and Causes of Preterm Birth. Lancet 371 (9606), 75–84. 10.1016/S0140-6736(08)60074-4 18177778 PMC7134569

[B28] Guo Y. You K. Qiao J. Zhao Y. Geng L. (2012). Bacterial Vaginosis Is Conducive to the Persistence of HPV Infection. Int. J. STD AIDS 23, 581–584. 10.1258/ijsa.2012.011342 22930296

[B29] Hammerschlag M. R. Alpert S. Onderdonk A. B. Thurston P. Drude E. McCormack W. M.. (1978a). Anaerobic Microflora of the Vagina in Children. Am. J. Obstet. Gynecol. 131 (8), 853–856. 10.1016/S0002-9378(16)33130-1 686083

[B30] Hammerschlag M. R. Alpert S. Rosner I. Thurston P. Semine D. McComb D.. (1978b). Microbiology of the Vagina in Children: Normal and Potentially Pathogenic Organisms. Pediatrics 62 (1), 57–62. 10.1016/s0002-9378(16)33130-1 98750

[B31] Hay P. E. Ugwumadu A. Chowns J. (1997). Sex, Thrush and Bacterial Vaginosis. Int. J. STD AID 8 (10), 603–608. 10.1258/0956462971918850 9310218

[B32] Hillier S. L. Lau R. J. (1997). Vaginal Microflora in Postmenopausal Women Who Have Not Received Estrogen Replacement Therapy. Clin. Infect. Dis. 25 (2), S123–S126. 10.1086/516221 9310650

[B33] Houdt R. Ma B. Bruisten S. M. Speksnijder A. G. C. L. Ravel J. de Vries H. J. C. (2018). Lactobacillus Iners-Dominated Vaginal Microbiota Is Associated With Increased Susceptibility to Chlamydia Trachomatis Infection in Dutch Women: A Case-Control Study. Sex Transm. Infect. 94 (2), 117–123. 10.1136/sextrans-2017-053133 28947665 PMC6083440

[B34] Huang H. Song L. Zhao W. (2014). Effects of Probiotics for the Treatment of Bacterial Vaginosis in Adult Women: A Meta-Analysis of Randomized Clinical Trials. Arch. Gynecol. Obstet. 289 (6), 1225–1234. 10.1007/s00404-013-3117-0 24318276

[B35] Jaisamrarn U. Triratanachat S. Chaikittisilpa S. Grob P. Prasauskas V. Taechakraichana N. (2013). Ultra-Low-Dose Estriol and Lactobacilli in the Local Treatment of Postmenopausal Vaginal Atrophy. Climacteric 16 (3), 347–355. 10.3109/13697137.2013.769097 23347400 PMC3786549

[B36] Keane F. E. Ison C. A. Taylor-Robinson D. (1997). A Longitudinal Study of the Vaginal Flora Over a Menstrual Cycle. Int. J. STD AIDS 8 (8), 489–494. 10.1258/0956462971920631 9259496

[B37] King C. C. Jamieson D. J. Wiener J. Cu-Uvin S. Klein R. S. Rompalo A. M.. (2011). Bacterial Vaginosis and the Natural History of Human Papillomavirus. Infect. Dis. Obstet. Gynecol. 2011, 319460. 10.1155/2011/319460 21869857 PMC3159014

[B38] Lee J. E. Lee S. Lee H. Song Y. M. Lee K. Han M. J.. (2013). Association of the Vaginal Microbiota With Human Papillomavirus Infection in a Korean Twin Cohort. PloS One 8 (5), e63514. 10.1371/journal.pone.0063514 23717441 PMC3661536

[B39] Lev-Sagie A. Goldman-Wohl D. Cohen Y. Dori-Bachash M. Leshem A. Mor U.. (2019). Vaginal Microbiome Transplantation in Women With Intractable Bacterial Vaginosis. Nat. Med. 25 (10), 1500–1504. 10.1038/s41591-019-0600-6 31591599

[B40] Liu M. B. Xu S. R. He Y. Deng G. H. Sheng H. F. Huang X. M.. (2013). Diverse Vaginal Microbiomes in Reproductive-Age Women With Vulvovaginal Candidiasis. PloS One 8 (11), e79812. 10.1371/journal.pone.0079812 24265786 PMC3827160

[B41] Lokken E. M. Richardson B. A. Kinuthia J. Mwinyikai K. Abdalla A. Jaoko W.. (2010). A Prospective Cohort Study of the Association Between Body Mass Index and Incident Bacterial Vaginosis. Sex Transm. Dis. 46 (1), 31–36. 10.1097/OLQ.0000000000000905 PMC6289672 30148757

[B42] Marrazzo J. M. (2006). A Persistent(Ly) Enigmatic Ecological Mystery: Bacterial Vaginosis. J. Infect. Dis. 193 (11), 1475–1477. 16652273 10.1086/503783

[B43] Marrazzo J. M. (2011). Interpreting the Epidemiology and Natural History of Bacterial Vaginosis: Are We Still Confused? Anaerobe 17 (4), 186–190. 10.1016/j.anaerobe.2011.03.016 21524714 PMC3155735

[B44] Martin D. H. (2012). The Microbiota of the Vagina and Its Influence on Women’s Health and Disease. Am. J. Med. Sci. 343 (1), 2–9. 10.1097/MAJ.0b013e31823ea228 22143133 PMC3248621

[B45] Miller E. A. Beasley D. E. Dunn R. R. Archie E. A. (2016). Lactobacilli Dominance and Vaginal pH: Why Is the Human Vaginal Microbiome Unique? Front. Microbiol. 7, 1936. 10.3389/fmicb.2016.01936 28008325 PMC5143676

[B46] Mirmonsef P. Modur S. Burgad D. Gilbert D. Golub E. T. French A. L.. (2015). Exploratory Comparison of Vaginal Glycogen and Lactobacillus Levels in Premenopausal and Postmenopausal Women. Menopause 22 (7), 702–709. 10.1097/GME.0000000000000397 25535963 PMC4476965

[B47] Mitchell C. M. Srinivasan S. Zhan X. Wu M. C. Reed S. D. Guthrie K. A.. (2017). Vaginal Microbiota and Genitourinary Menopausal Symptoms:a Cross-Sectional Analysis. Menopause 24 (10), 1160–1166. 10.1097/GME.0000000000000904 28640154 PMC5607086

[B48] Mitra A. MacIntyre D. A. Lee Y. S. Smith A. Marchesi J. R. Lehne B.. (2015). Cervical Intraepithelial Neoplasia Disease Progression Is Associated With Increased Vaginal Microbiome Diversity. Sci. Rep. 5, 16865. 10.1038/srep16865 26574055 PMC4648063

[B49] Mitra A. MacIntyre D. A. Marchesi J. R. Lee Y. S. Bennett P. R. Kyrgiou M. (2016). The Vaginal Microbiota, Human Papillomavirus Infection and Cervical Intraepithelial Neoplasia: What Do We Know and Where Are We Going Next? Microbiome 4 (1), 58. 10.1186/s40168-016-0203-0 27802830 PMC5088670

[B50] Muhleisen A. L. Herbst-Kralovetz M. M. (2016). Menopause and the Vaginal Microbiome. Maturitas 91, 42–50. 10.1016/j.maturitas.2016.05.015 27451320

[B51] Nardini P. Ñahui Palomino R. A. Parolin C. Laghi L. Foschi C. Cevenini R.. (2016). Lactobacillus Crispatus Inhibits the Infectivity of Chlamydia Trachomatis Elementary Bodies, In Vitro Study. Sci. Rep. 6, 29024. 10.1038/srep29024 27354249 PMC4926251

[B52] Nugent R. P. Krohn M. A. Hillier S. L. (1991). Reliability of Diagnosing Bacterial Vaginosis is Improved by a Standardized Method of Gram Stain Interpretation. J. Clin. Microbiol. 29 (2), 297–301. 10.1128/jcm.29.2.297-301.1991 1706728 PMC269757

[B53] Panduru M. Panduru N. M. Sălăvăstru C. M. Tiplica G. S. (2015). Probiotics and Primary Prevention of Atopic Dermatitis: A Meta-Analysis of Randomized Controlled Studies. J. Eur. Acad. Dermatol. Venereol. 29 (2), 232–242. 10.1111/jdv.12496 24698503

[B54] Pasolli E. De Filippis F. Mauriello I. E. Cumbo F. Walsh A. M. Leech J.. (2020). Large-Scale Genome-Wide Analysis Links Lactic Acid Bacteria From Food With the Gut Microbiome. Nat. Commun. 11 (1), 2610. 10.1038/s41467-020-16438-8 32451391 PMC7248083

[B55] Petricevic L. Unger F. M. Viernstein H. Kiss H. (2008). Randomized, Double-Blind, Placebo-Controlled Study of Oral Lactobacilli to Improve the Vaginal Flora of Postmenopausal Women. Eur. J. Obstet. Gynecol. Reprod. Biol. 141 (1), 54–57. 10.1016/j.ejogrb.2008.06.003 18701205

[B56] Petrova M. I. Reid G. Vaneechoutte M. Lebeer S. (2017). Lactobacillus Iners: Friend or Foer. Trends Microbiol. 25 (3), 182–191. 10.1016/j.tim.2016.11.007 27914761

[B57] Priestley C. J. Jones B. M. Dhar J. Goodwin L. (1997). What Is Normal Vaginal Flora? Genitourin Med. 73 (1), 23–28. 10.1136/sti.73.1.23 9155551 PMC1195755

[B58] Pybus V. Onderdonk A. B. (1999). Microbial Interactions in the Vaginal Ecosystem, With Emphasis on the Pathogenesis of Bacterial Vaginosis. Microbes Infect. 1, 285–292. 10.1016/S1286-4579(99)80024-0 10602662

[B59] Randis T. M. Ratner A. J. (2019). Gardnerella and Prevotella: Co-Conspirators in the Pathogenesis of Bacterial Vaginosis. J. Infect. Dis. 220 (7), 1085–1088. 10.1093/infdis/jiy705 30715397 PMC6736359

[B60] Ravel J. Gajer P. Abdo Z. Schneider G. M. Koenig S. S. K. McCulle S. L.. (2011). Vaginal Microbiome of Reproductive-Age Women. PNAS 15 (108 Suppl 1), 4680–4687. 10.1073/pnas.1002611107 PMC3063603 20534435

[B61] Redondo-Lopez V. Cook R. L. Sobel J. D. (1990). Emerging Role of Lactobacilli in the Control and Maintenance of the Vaginal Bacterial Microflora. Rev. Infect. Dis. 12 (5), 856–872. 10.1093/clinids/12.5.856 2237129

[B62] Relman D. A. (2015). The Human Microbiome and the Future Practice of Medicine. JAMA 314 (11), 1127–1128. 10.1001/jama.2015.10700 26372576

[B63] Ribeiro A. N. Santana Monteiro N. E. Gueldini de Moraes A. V. Costa-Paiva L. H. Orcesi Pedro A. (2018). Can the Use of Probiotics in Association With Isoflavone Improve the Symptoms of Genitourinary Syndrome of Menopause? Results Randomized Controlled Trial. Menopause 26 (6), 643–652. 10.1097/GME.0000000000001279 30531444

[B64] Romero R. Dey S. K. Fisher S. J. (2014). Preterm Labor: One Syndrome, Many Causes. Science 345 (6198), 760–765. 10.1126/science.1251816 25124429 PMC4191866

[B65] Romero R. Hassan S. S. Gajer P. Tarca A. L. Fadrosh D. W. Bieda J.. (2014). The Vaginal Microbiota of Pregnant Women Who Subsequently Have Spontaneous Preterm Labor and Delivery and Those With a Normal Delivery at Term. Microbiome 2, 18. 10.1186/2049-2618-2-18 24987521 PMC4066267

[B66] Romero R. Hassan S. S. Gajer P. Tarca A. L. Fadrosh D. W. Nikita L.. (2014). The Composition and Stability of the Vaginal Microbiota of Normal Pregnant Women Is Different From That of non-Pregnant Women. Microbiome 2 (1), 4. 10.1186/2049-2618-2-18 24484853 PMC3916806

[B67] Savino F. Cordisco L. Tarasco V. Palumeri E. Calabrese R. Oggero R.. (2010). Lactobacillus Reuteri DSM 17938 in Infantile Colic: A Randomized, Double-Blind, Placebo-Controlled Trial. Pediatrics 126 (3), e526–e533. 10.1542/peds.2010-0433 20713478

[B68] Schwebke J. R. Richey C. M. Weiss H. L. (1999). Correlation of Behaviors With Microbiological Changes in Vaginal Flora. J. Infect. Dis. 180 (5), 1632–1636. 10.1086/315065 10515826

[B69] Sobel J. D. (1999). Is There a Protective Role for Vaginal Flora? Curr. Infect. Dis. Rep. 1 (4), 379–383. 10.1007/s11908-999-0045-z 11095812

[B70] Srinivasan S. Liu C. Mitchell C. M. Fiedler T. L. Thomas K. K. Agnew K. J.. (2010). Temporal Variability of Human Vaginal Bacteria and Relationship With Bacterial Vaginosis. PloS One 5 (4), e10197. 10.1371/journal.pone.0010197 20419168 PMC2855365

[B71] Stackebrandt E. Goebel B. M. (1994). Taxonomic Note: A Place for DNA-DNA Reassociation And 16S rRNA Sequence Analysis in the Present Species Definition in Bacteriology. Int. J. Syst. Bact. 44 (4), 846–849. 10.1099/00207713-44-4-846

[B72] Tamburini S. Shen N. Wu H. C. Jose C. Clemente J. C. (2016). The Microbiome in Early Life: Implications for Health Outcomes. Nat. Med. 22 (7), 713–722. 10.1038/nm.4142 27387886

[B73] Thoma M. E. Gray R. H. Kiwanuka N. Aluma S. Wang M. C. Sewankambo N.. (2011). Longitudinal Changes in Vaginal Microbiota Composition Assessed by Gram Stain Among Never Sexually Active Pre- and Postmenarcheal Adolescents in Rakai, Uganda. J. Pediatr. Adolesc. Gynecol. 24, 42–7. 10.1016/j.jpag.2010.07.002 PMC2988102 20709584

[B74] Turnbaugh P. J. Ley R. E. Hamady M. Fraser-Liggett C. M. Knight R. Gordon J. I. (2007). The Human Microbiome Project. Nature 449, 804–810. 10.1038/nature06244 17943116 PMC3709439

[B75] Underhill D. M. (2013). Striking a Balance: Fungal Commensalism Versus Pathogenesis. Curr. Opin. Microbiol. 16 (3), 366–373. 10.1016/j.mib.2013.05.004 23756050 PMC3742553

[B76] Vasquez A. Jakobsson T. Ahrné S. Forsum U. Maeyolin G. (2002). Vaginal Lactobacillus Flora of Healthy Swedish Women. J. Clin. Microbiol. 40 (8), 2746–2749. 10.1128/JCM.40.8.2746-2749.2002 12149323 PMC120688

[B77] Vylkova S. Carman A. J. Danhof H. A. Collette J. R. Zhou H. Lorenz M. C. (2011). The Fungal Pathogen Candida Albicans Autoinduces Hyphal Morphogenesis by Raising Extracellular Ph. MBio 2 (3), e00055. 10.1128/mBio.00055-11 21586647 PMC3101780

[B78] WHO (2018). Report on Global Sexually Transmitted Infection Surveillance. (World Health Organization).

[B79] Witkin S. S. Linhares I. M. Giraldo P. (2007). Bacterial Flora of the Female Genital Tract: Function and Immune Regulation. Best Pract. Res. Clin. Obstet. Gynaecol. 21 (3), 347–354. 10.1016/j.bpobgyn.2006.12.004 17215167

[B80] Workowski K. A. Berman S. (2010). Centers for Disease Control and Prevention (CDC). (2010) Sexually Transmitted Diseases Treatment Guidelines. MMWR Recomm. Rep. 59 (RR-12), 1–110. 21160459

[B81] Wu W. Liao Q. Liu Z. (2017). Analysis of the Vaginal Microecology in Patients With Severe Vulvovaginal Candidiasis. BioMed. Res. 28 (1), 118–121.

[B82] Xie H. Y. Feng D. Wei D. M. Mei L. Chen H. Wang X.. (2017). Probiotics for Vulvovaginal Candidiasis in Non-Pregnant Women. Cochrane Database Syst. Rev. 11 (11), CD010496. 10.1002/14651858.CD010496.pub2 29168557 PMC6486023

[B83] Zhou X. Brown C. J. Abdo Z. Davis C. C. Hansmann M. A. Joyce P.. (2007). Differences in the Composition of Vaginal Microbial Communities Found in Healthy Caucasian and Black Women. ISME J. 1 (2), 121–133. 10.1038/ismej.2007.12 18043622

[B84] Zhou X. Westman R. Hickey R. Hansmann M. A. Kennedy C. Osborn T. W.. (2009). Vaginal Microbiota of Women With Frequent Vulvovaginal Candidiasis. Infect. Immun. 77 (9), 4130–4135. 10.1128/IAI.00436-09 19528218 PMC2738030

[B85] Ziklo N. Huston W. M. Taing K. Katouli M. Timms P. (2016). In Vitro Rescue of Genital Strains of Chlamydia Trachomatis From Interferon-γ and Tryptophan Depletion With Indole-Positive, But Not Indole-Negative Prevotella Spp. BMC Microbiol. 16 (1), 286. 10.1186/s12866-016-0903-4 27914477 PMC5135834

